# Is Standing Long Jump Performance More Strongly Associated with Health-Related Outcomes than Maximal Isometric Handgrip Strength in Adolescents?

**DOI:** 10.3390/children13030314

**Published:** 2026-02-24

**Authors:** Felipe Montalva-Valenzuela, Antonio Castillo-Paredes, Yeny Concha-Cisternas, Exal Garcia-Carrillo, Natalia Escobar Ruiz, Rodrigo Yañez-Sepúlveda, Iván Molina-Márquez, Eduardo Guzmán-Muñoz

**Affiliations:** 1 Escuela de Entrenador en Actividad Física y Deporte, Facultad de Ciencias Humanas, Universidad Bernardo O’Higgins, Santiago 8370040, Chile; 2 Grupo AFySE, Investigación en Actividad Física y Salud Escolar, Escuela de Pedagogía en Educación Física, Facultad de Educación, Universidad de Las Américas, Santiago 8370040, Chile; acastillop85@gmail.com; 3 Escuela de Kinesiología, Facultad de Salud, Universidad Santo Tomás, Talca 3460000, Chile; 4 Vicerrectoría de Investigación e Innovación, Universidad Arturo Prat, Iquique 1100000, Chile; 5 Department of Physical Activity Sciences, Faculty of Education Sciences, Universidad Católica del Maule, Talca 3480112, Chile; exal.garcia@gmail.com; 6 Department of Physical Activity Sciences, Universidad de Los Lagos, Osorno 5290000, Chile; 7 Facultad de Educación y Ciencias Sociales, Universidad Andrés Bello, Viña del Mar 2200055, Chile; rodrigo.yanez.s@unab.cl; 8 School of Medicine, Universidad Espíritu Santo, Samborondón 092301, Ecuador; 9 Escuela de Educación Física, Facultad de Educación, Universidad Adventista de Chile, Chillán 3780000, Chile; 10 Programa Doctorado en Ciencias de la Actividad Física, Universidad Católica del Maule, Talca 3460000, Chile; 11 Pedagogía en Educación Física, Facultad de Educación, Universidad Autónoma de Chile, Talca 3460000, Chile

**Keywords:** standing long jump, maximal isometric handgrip strength, adolescents, muscular fitness, health-related outcomes

## Abstract

Background: Muscular fitness is an important marker of current and future health in adolescents. However, comparisons are lacking that show which tests are most consistently associated with health-related outcomes during adolescence. Objective: This study aimed to analyze whether performance in the SLJ is more strongly associated with health-related outcomes than maximal isometric handgrip strength (MIHS) in secondary school students in Chile. Methods: A cross-sectional study was conducted in 113 adolescents (77 males and 36 females, mean age 15.90 ± 1.77). Muscular fitness was assessed using the SLJ and MIHS tests. Health-related outcomes included body mass index (BMI), physical activity level, aerobic capacity, sprint performance, flexibility, sleep quality, and psychological well-being. Pearson correlation analyses and multiple linear regression models adjusted for sex and age were performed to examine associations between muscular fitness tests and health-related outcomes. Results: SLJ showed moderate-to-strong associations with several health-related outcomes, including physical activity (r = 0.42, *p* < 0.001), aerobic capacity (r = 0.60, *p* < 0.001), sprint performance (r = −0.74, *p* < 0.001), and body mass index (r = −0.30, *p* = 0.001). In contrast, handgrip strength demonstrated weaker and less consistent associations. In adjusted regression models, SLJ remained a significant predictor of most outcomes (β range: 0.27–0.56, *p* < 0.05), whereas handgrip strength provided limited additional explanatory value. Conclusions: SLJ appears to be a more sensitive and consistent indicator of health-related outcomes in adolescents than MIHS. These findings support the use of SLJ as a practical, low-cost, and easily implementable tool for health and fitness screening in school and community settings.

## 1. Introduction

Muscular fitness is considered an important marker of current health in children and adolescents [1,2]. In this population, low or insufficient muscular strength has been associated with multiple cardiovascular risk factors, premature diseases, and even suicide [3], as well as with higher levels of stress [4], greater indicators of depression [5], anxiety [6] and increased engagement in health-risk behaviors such as tobacco and alcohol consumption and episodes of drunkenness [7]. Conversely, adequate muscular fitness has been associated with better mental health [8]; in adults, a positive association with higher self-esteem has been observed [9], while in adolescents, it has been related to better health-related quality of life [10]. 

Muscular fitness is composed of muscular strength, explosive power, and endurance [11], and it can be assessed using different field-based tests. The standing long jump (SLJ) is a widely used test as an indicator of muscular fitness across different age groups [12,13] and is characterized by being practical, low-cost, and easy to interpret, in addition to being validated in different populations [14,15]. The SLJ reflects lower-limb explosive strength and whole-body coordination, capturing dynamic force production and neuromuscular integration. In turn, maximal isometric handgrip strength (MIHS) is a direct and simple test [16], also validated in different populations [17,18]. MIHS primarily evaluates upper-limb isometric strength and provides a measure of muscular capacity that is related to health outcomes in adolescents, also reflecting overall muscular strength [19]. It is easily comparable across studies and has predictive clinical value [20].

Adolescence is a key stage for the establishment of healthy habits, including physical activity [21]. However, despite the extensive evidence on the benefits of physical activity and muscular fitness, children and adolescents exhibit low levels of physical activity, and a large proportion of daily time is devoted to sedentary behaviors such as studying, watching television, or using social media [22]. It is estimated that, worldwide, nearly 80% of the adolescent population is insufficiently active, and many spend at least two hours per day on screen-based activities [21]. In addition, there is increased exposure to factors associated with stress, anxiety, and sleep disturbances [23,24,25], which reinforces the need for simple and sensitive indicators to allow early identification of health risk profiles in adolescent populations.

In addition to the above, health-related outcomes are increasingly being studied, with a growing emphasis on health and well-being [26]. Nevertheless, there is limited evidence directly comparing which muscular fitness tests are more consistently associated with different health outcomes during adolescence. Until now, no study has comprehensively compared SLJ and MIHS within the same adolescent sample across physical, psychological, and lifestyle-related outcomes, leaving a gap in understanding their relative predictive value. From an applied perspective, identifying the muscular test with the strongest and most consistent association with health indicators could optimize assessment processes in school and community settings, where time constraints, limited resources, and methodological simplicity are key factors. In this context, the present study aimed to analyze whether performance in the SLJ is more strongly associated with health-related outcomes than MIHS in secondary school students in Chile.

## 2. Materials and Methods

### 2.1. Study Design

This study adopted a quantitative, descriptive–correlational methodology using a cross-sectional observational design [27]. Ethical approval was granted by the Ethics Committee of Universidad Santo Tomás (Approval No. 23–27), and all procedures complied with the Declaration of Helsinki. Reporting of the study adhered to the Strengthening the Reporting of Observational Studies in Epidemiology (STROBE) guidelines [28].

### 2.2. Participants

The study included a non-probabilistic convenience sample of 113 adolescents (77 males and 36 females) aged 14–18 years (mean age: 15.90 ± 1.77), recruited from a subsidized private secondary school in the Metropolitan Region of Chile due to accessibility and feasibility considerations. Participants were required to meet the following inclusion criteria: (i) enrollment in secondary education; (ii) voluntary participation supported by informed assent and written parental consent; (iii) absence of musculoskeletal injuries or medical contraindications to physical fitness testing. Adolescents reporting acute pain or injury within the previous three months were excluded from the analyses.

An a priori sample size calculation was conducted to ensure adequate statistical power for the planned multiple linear regression analyses. The calculation was performed using G*Power software (version 3.1.9.7) for a fixed-model regression design testing the deviation of R^2^ from zero. The primary reference model corresponded to Model 3, which included four predictors (age, sex, standing long jump, and handgrip strength). Based on effect sizes reported in the previous literature [9], a conservative medium effect size was assumed (f^2^ = 0.15). With a significance level of α = 0.05, statistical power of 80% (1 − β = 0.80), and four predictors, the minimum required sample size was estimated at 85 participants.

### 2.3. Procedures

Data collection was structured into three main domains: anthropometric measurements, physical fitness assessments, and health-related outcomes. All evaluations were conducted in the school gymnasium under standardized environmental conditions (morning hours, temperature at 20–23 °C), with participants wearing standardized sports clothing. Assessments were conducted on non-consecutive days, with at least 48 h between sessions, to minimize fatigue and ensure recovery.

On the first day, participants underwent anthropometric assessments and a familiarization session. All testing protocols were explained and demonstrated by the research staff. The second day was dedicated to physical fitness assessments, including the SLJ and MIHS tests. In addition, complementary physical fitness evaluations were conducted, consisting of the Cooper 12 min run test, the 20 m sprint test, the sit-and-reach test, and the scratch test. On the third day, health-related outcomes were assessed through validated questionnaires, including the Physical Activity Questionnaire for Adolescents (PAQ-A), the Pittsburgh Sleep Quality Index (PSQI), and the Depression, Anxiety, and Stress Scale–21 (DASS–21), administered individually in the classroom using participants’ smartphones via Google Forms, under researcher supervision.

### 2.4. Physical Fitness Assessments

Prior to all physical fitness evaluations, participants performed a standardized general warm-up lasting approximately 5 min, consisting of joint mobility exercises and light jogging to reduce injury risk and optimize performance.

Lower-limb explosive muscle strength was assessed using the SLJ test following standardized procedures [29]. The test was conducted indoors on a non-slip surface. Participants started from an upright position behind a marked take-offline, with feet parallel and shoulder-width apart, and performed three maximal horizontal jumps using both legs simultaneously with free arm movement. Jump distance (cm) was measured from the take-offline to the closest heel contact using a flexible steel tape. A minimum of one minute of passive rest was provided between attempts, and the longest distance achieved was retained for analysis.

Upper-limb muscle strength was assessed through MIHS using a digital dynamometer (Camry EH101, Sensun Weighing Apparatus Group Ltd., Guangdong, China). Participants stood upright with the elbow flexed at 90°, the forearm in a neutral position, and the arm held close to the trunk. The handle was adjusted to each participant’s hand size, and they were instructed to perform three maximal contractions of approximately three seconds, avoiding compensatory movements. The highest value (kg) obtained was recorded for analysis [18].

Cardiorespiratory fitness was assessed using the Cooper 12 min run test [30], which requires participants to cover the greatest possible distance within 12 min. The test was conducted on a 400 m school athletic track, and performance was recorded as the total distance covered, calculated based on completed laps and additional distance measured using cones placed at 100 m intervals along the track.

Speed performance was evaluated using the 20 m sprint test [31]. Participants started from a standing position behind a clearly marked line and, upon an auditory signal (whistle), sprinted maximally over a distance of 20 m. Sprint time was recorded in seconds at the moment the participant crossed the finish line.

Flexibility was assessed using two complementary tests: the sit-and-reach test to evaluate hamstring and lower-back flexibility [32], and the back scratch test to assess shoulder flexibility [33]. For the sit-and-reach test, participants sat with knees fully extended and reached forward toward (or beyond) the toes; the best distance achieved (cm) was recorded. For the back scratch test, participants attempted to touch or overlap the middle fingers of both hands behind the back, and the distance between fingers (positive or negative, in cm) was recorded, with the best attempt used for analysis.

### 2.5. Health-Related Outcomes

Health-related outcomes included body mass index (BMI), sleep quality, physical activity level, and psychological well-being. BMI was calculated as body weight (kg) divided by height squared (m^2^), using measurements obtained with a calibrated digital scale and a wall-mounted stadiometer (Seca; accuracy 0.1 cm).

Sleep quality was assessed using the PSQI [34], a validated questionnaire composed of seven components evaluating sleep duration, latency, efficiency, disturbances, use of sleep medication, and daytime dysfunction. Global PSQI scores range from 0 to 21 points, with scores ≤ 5 indicating good sleep quality and scores > 5 reflecting poor sleep quality.

Physical activity levels were evaluated using the PAQ-A [35], which assesses moderate-to-vigorous physical activity over the previous seven days across school, leisure, and sports contexts, yielding a final score ranging from 1 (low activity) to 5 (high activity).

Psychological well-being was assessed using the DASS-21 [36], consisting of 21 items distributed across three subscales with seven items each. Responses were rated on a 4-point Likert scale, and average scores were calculated for each subscale, with higher values indicating greater levels of depressive symptoms, anxiety, or stress.

### 2.6. Statistical Analysis

Descriptive statistics are presented as mean ± standard deviation for continuous variables. Normality of the data distribution was evaluated using the Shapiro–Wilk test. Bivariate correlations between neuromuscular performance tests (SLJ and MIHS) and health-related outcomes were examined using Pearson’s correlation coefficients. The strength of the associations was interpreted according to conventional thresholds, with correlation coefficients < 0.40 considered weak, values between 0.40 and 0.69 considered moderate, and values ≥ 0.70 considered strong.

Multiple linear regression analyses were conducted to examine the associations of SLJ and MIHS with health-related outcomes. For each outcome, three regression models were tested: Model 1 included sex, age, and SLJ; Model 2 included sex, age, and MIHS; and Model 3 included sex, age, SLJ, and MIHS simultaneously. Standardized beta coefficients (β), confidence intervals (95% CI), coefficients of determination (R^2^), and *p*-values are reported for all regression models. The additional explained variance of the combined model (Model 3) compared with the best single-test model (Model 1 or Model 2) was examined to quantify the gain in explained variance when both SLJ and MIHS were included simultaneously, thereby indicating whether the two tests provided complementary information or whether one test was redundant once the other was considered. Regression assumptions, including linearity, homoscedasticity, and absence of multicollinearity, were verified prior to analysis. All statistical analyses were performed using Jamovi statistical software (version 2.5), and statistical significance was set at *p* < 0.05.

## 3. Results

A total of 113 adolescents participated in the study, comprising 77 males (68.1%) and 36 females (31.9%). Descriptive characteristics of the total sample and stratified by sex are presented in Table 1. Descriptive statistics for SLJ, MIHS, and health-related outcomes are presented in Table 2.

**Figure 1:**
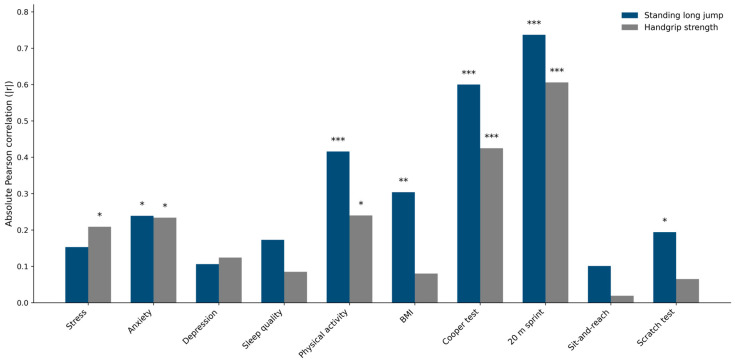
Comparison of the strength of correlations (absolute Pearson’s r values) between SLJ and MIHS with health-related outcomes in adolescents (*n* = 113). Asterisks indicate statistically significant correlations (* *p* < 0.05, ** *p* < 0.01, *** *p* < 0.001).

The results of the correlation analyses are presented in Figure 1. Overall, SLJ performance showed stronger and more consistent associations with health-related outcomes compared with MIHS. In relation to psychological health indicators, SLJ was significantly associated with anxiety symptoms (r = −0.24, *p* = 0.011), whereas no significant associations were observed with stress or depressive symptoms. The association between SLJ and sleep quality showed a statistical trend (r = −0.17, *p* = 0.067).

Regarding physical activity and body composition, SLJ was significantly associated with physical activity level (r = 0.42, *p* < 0.001) and was inversely associated with BMI (r = −0.30, *p* = 0.001). With respect to health-related physical fitness, significant associations were observed with aerobic capacity assessed by the Cooper test (r = 0.60, *p* < 0.001) and with sprint performance in the 20 m sprint test (r = −0.74, *p* < 0.001). Finally, SLJ was significantly associated with scratch test performance (r = 0.19, *p* = 0.040), while no significant association was observed with the sit-and-reach test.

In contrast, MIHS showed significant associations with stress (r = −0.21, *p* = 0.026) and anxiety (r = −0.23, *p* = 0.013), whereas no significant associations were observed with depressive symptoms or sleep quality. MIHS was significantly associated with physical activity level (r = 0.24, *p* = 0.011) but was not significantly associated with BMI. Regarding physical fitness outcomes, MIHS showed significant associations with aerobic capacity (r = 0.43, *p* < 0.001) and 20 m sprint performance (r = −0.61, *p* < 0.001), while no significant associations were observed with flexibility-related outcomes.

The results of the multiple linear regression analyses are presented in Table 3, which includes only outcomes showing at least one statistically significant regression model after adjustment for sex and age, including BMI, physical activity level, aerobic capacity assessed by the Cooper test, 20 m sprint performance, and scratch test performance. No statistically significant regression models were observed for stress, anxiety, depression, sleep quality, or flexibility assessed by the sit-and-reach test after adjustment for sex and age. Overall, SLJ demonstrated stronger and more consistent associations with health-related outcomes than MIHS.

For BMI, both SLJ and MIHS were independently associated with BMI when included separately in the regression models. However, when both tests were entered simultaneously (Model 3), SLJ (β = −0.469, *p* < 0.001) and MIHS (β = 0.485, *p* < 0.001) remained significant predictors, increasing the explained variance from approximately 10% in the single-test models to 22.5% in the combined model. This corresponds to an additional 12.3% of explained variance, indicating that the combined assessment of lower- and upper-limb strength provided complementary information for BMI beyond sex and age.

Regarding physical activity level (PAQ-A score), SLJ was significantly associated with physical activity in both Model 1 (β = 0.405, *p* < 0.001) and Model 3 (β = 0.397, *p* < 0.001), whereas MIHS was not significant either when analyzed alone or when combined with SLJ. The inclusion of MIHS did not increase the explained variance, indicating that SLJ alone captured the relevant variance in physical activity.

A similar pattern was observed for aerobic capacity assessed by the Cooper test. SLJ showed a strong association in Model 1 (β = 0.402, *p* < 0.001) and remained significant in Model 3 (β = 0.408, *p* < 0.001), while MIHS was not significantly associated in any model. Accordingly, the combined model did not improve the explained variance beyond that of the SLJ-only model, suggesting that MIHS offers no additional predictive value for aerobic capacity.

## 4. Discussion

This study aimed to analyze whether performance in the SLJ is more strongly associated with health-related outcomes than MIHS in secondary school students in Chile. Given the cross-sectional design of the study, the results should be interpreted as associations rather than evidence of causality. Nevertheless, the consistency and magnitude of the associations observed across several health-related outcomes provide valuable insight into the relative explanatory capacity of SLJ and MIHS during adolescence.

The main finding of the present study is that SLJ emerged as a more sensitive and consistent indicator of health-related outcomes in adolescents compared with MIHS. Across correlation and regression analyses adjusted for sex and age, SLJ showed stronger associations with BMI, physical activity level, aerobic capacity, sprint performance, and flexibility assessed by the scratch test, whereas MIHS demonstrated a more limited and outcome-specific predictive value. Notably, the magnitude of these associations ranged from moderate to strong for SLJ across several outcomes, whereas associations involving MIHS were generally weaker and less consistent, indicating meaningful differences in the explanatory capacity of both tests. Importantly, for several outcomes, adding MIHS to the regression models did not substantially improve their explanatory capacity beyond that achieved by SLJ alone. This finding suggests that some of the variance explained by MIHS may coincide with that captured by lower-limb explosive performance, reinforcing the broader explanatory value of the SLJ in this population.

These results are in line with previous studies reporting significant associations between lower-limb explosive performance and indicators of cardiometabolic health, body composition, and cardiorespiratory fitness in youth populations [37]. Although handgrip strength has demonstrated clinical and prognostic value in both pediatric and adult populations [38], its associations with broader lifestyle and psychosocial indicators during adolescence appear to be more variable, which may partially explain the differences observed in our study.

From a physiological perspective, the stronger associations observed for SLJ compared with MIHS may be explained by the greater integrative demands of lower-limb explosive performance. The SLJ requires the coordinated contribution of muscle strength, power, neuromuscular activation, intermuscular coordination, and postural control, as well as an effective transfer of force through the kinetic chain [39,40]. These demands are particularly relevant during adolescence, a developmental period characterized by rapid changes in body size, muscle mass, and neuromuscular maturation, which increase the requirements for coordination and motor control [41,42]. In adolescents, this task reflects not only maximal force production but also motor control efficiency and the ability to rapidly generate force. These characteristics are closely linked to habitual physical activity levels, cardiorespiratory fitness, and body composition. In contrast, MIHS predominantly reflects isolated upper-limb force capacity and is less dependent on whole-body coordination or dynamic movement patterns [17]. Therefore, it may be less sensitive to developmental changes in neuromuscular maturation and movement efficiency during adolescence.

Beyond physiological mechanisms, contextual factors may also have influenced the observed associations. The sample was drawn from a single school using convenience sampling, and regional, socioeconomic, environmental, and educational characteristics may shape adolescents’ physical activity levels and motor development. Differences in access to sports facilities, quality of physical education classes, and opportunities for organized sport participation may affect fitness outcomes. Importantly, specific information regarding participants’ sport participation was not collected, which limits our ability to account for its potential influence on performance and health-related indicators. Therefore, caution is warranted when generalizing these findings to adolescents from other regions or socioeconomic contexts.

Taken together, these findings indicate that SLJ and MIHS should not be considered interchangeable indicators of muscular fitness in adolescents. While both tests provide information related to general strength capacity, the SLJ appears to offer complementary and functionally relevant information that is not fully captured by MIHS. Specifically, SLJ captures dynamic aspects of lower-limb performance that appear to be closely linked to several health-related indicators during adolescence. Consequently, exclusive reliance on handgrip strength may underestimate key dimensions of physical function that are closely associated with multiple health-related outcomes in this population.

These findings are consistent with previous research highlighting the SLJ as a good indicator of overall muscular fitness and health-related quality of life in adolescents [1,43]. However, beyond aligning with prior evidence, the present study extends existing knowledge by demonstrating that SLJ shows systematically stronger associations with a broad range of health-related outcomes than MIHS.

From a practical perspective, the associations observed for the SLJ have important implications for health monitoring and decision-making in school-aged children, in educational and community settings. Due to its simplicity, low cost, and minimal equipment requirements, the SLJ emerges as a suitable tool for monitoring adolescent health. In school-based physical education assessments and public health screening programs, incorporating the SLJ may facilitate early identification of adolescents at potential health risk. The SLJ may also provide insight into key aspects of adolescent health, including physical activity, aerobic capacity, and body composition. Incorporating the SLJ into routine school-based assessments could therefore offer a broader perspective on adolescent health than relying solely on isolated strength measures such as MIHS.

### 4.1. Limitations

This study is not without limitations. Among them is the sample selection, which was based on convenience sampling. There is also an imbalance in the number of male and female participants, so we recommend interpreting these results with appropriate restraint, as we cannot definitively state that they are the same for men and women.

The use of self-report questionnaires can also generate some uncertainty, as it often depends on reading comprehension, participants’ honesty, attention, concentration, and other external factors that are often beyond our control.

It was not possible to fully control external fatigue from other activities (poor sleep, excessive study hours, or participation in other sports). Although participants were asked to take care of themselves during the week, there is no guarantee that they did so. Furthermore, the motivational component of physical tests is always important to consider. Additionally, information regarding participants’ sport participation was not collected, which may influence performance and health-related outcomes. Finally, and importantly, relationships do not establish causality, so we recommend interpreting the results with appropriate restraint.

### 4.2. Future Lines of Investigation

In future lines of research, we recommend including additional upper-body muscular fitness tests, such as the push-up test. Given the greater coordination demands of this exercise, it may represent a better comparison measure.

We also recommend increasing the sample size, particularly regarding the number of participants, the proportion of female participants, and the use of randomized procedures.

Another interesting point would be to consider the participants’ socioeconomic status, recreational or leisure activities, available spaces or open areas for recreation and whether they live in an apartment or a house.

Finally, we recommend incorporating body composition variables, as indicators such as muscle mass or fat mass may provide more informative insights than body weight alone.

### 4.3. Practical Recommendations

Prioritizing the use of the SLJ over the MIHS can be a viable option, as it provides information on muscle fitness and overall health. Due to its ease of administration, it can be systematically incorporated into various contexts, including schools, communities, and even clinical settings, where time, resources, and feasibility are key considerations.

## 5. Conclusions

The SLJ is a more sensitive and consistent indicator of health-related outcomes in adolescents compared to the MIHS. These findings support the SLJ as a practical, readily available, and cost-effective tool for general health and fitness indicators, with potential value as a simple screening measure for identifying adolescents at higher health risk.

Additionally, the SLJ showed associations with BMI, physical activity level, aerobic capacity, sprint performance, and flexibility as assessed by the scratch test.

## Figures and Tables

**Table 1:** Descriptive characteristics of the study sample according to sex.

Variable	Total (*n* = 113)	Males (*n* = 77)	Females (*n* = 36)
Age (years)	16.03 ± 1.10	16.08 ± 1.10	15.92 ± 1.11
Weight (kg)	62.85 ± 9.03	64.81 ± 8.73	58.66 ± 8.29
Height (cm)	167.19 ± 8.42	171.22 ± 6.03	158.58 ± 6.02
BMI (kg/m^2^)	22.49 ± 2.87	22.09 ± 2.71	23.32 ± 3.05

BMI, body mass index.

**Table 2:** Descriptive characteristics of SLJ, MIHS, and health-related outcomes according to sex.

Variable	Total (*n* = 113)	Males (*n* = 77)	Females (*n* = 36)
SLJ (cm)	181.99 ± 30.56	194.26 ± 25.45	155.75 ± 23.30
MIHS (kg)	32.88 ± 7.96	36.02 ± 7.27	26.16 ± 4.48
Stress (score)	9.65 ± 4.20	8.91 ± 4.08	11.22 ± 4.07
Anxiety (score)	7.35 ± 4.73	6.31 ± 4.13	9.58 ± 5.22
Depression (score)	7.83 ± 4.99	7.35 ± 4.70	8.86 ± 5.47
Sleep quality (score)	6.81 ± 3.78	6.16 ± 3.45	8.20 ± 4.11
Physical activity (PAQ-A score)	2.58 ± 0.76	2.71 ± 0.74	2.28 ± 0.72
Cooper test (laps)	22.38 ± 5.56	24.55 ± 4.22	17.75 ± 5.28
20 m sprint (s)	3.77 ± 0.42	3.59 ± 0.33	4.16 ± 0.33
Sit-and-reach (cm)	5.43 ± 9.63	3.64 ± 8.66	9.28 ± 10.58
Scratch test (cm)	5.50 ± 5.20	5.63 ± 5.05	5.21 ± 5.56

**Table 3:** Significant multiple linear regression models examining the associations of Standing long jump (SLJ) and Maximal isometric handgrip strength (MIHS) with health-related outcomes in adolescents, adjusted for sex and age.

Outcome	Model	R^2^	β SLJ [CI 95%] (*p*)	β MIHS [CI 95%] (*p*)	ΔR^2^ (Model 3)
BMI (Kg/m^2^)	Model 1 (SLJ)	0.094	−0.285 [−0.510, −0.060] (0.013)		0.123
	Model 2 (MIHS)	0.102		0.306 [0.080, 0.532] (0.008)	
	Model 3 (MIHS + SLJ)	0.225	−0.469 [−0.733, −0.205] (<0.001)	0.485 [0.211, 0.759] (<0.001)	
Physical activity (PAQ-A score)	Model 1 (SLJ)	0.228	0.405 [0.177, 0.633] (<0.001)		<0.001
	Model 2 (MIHS)	0.141		0.174 [−0.044, 0.392] (0.117)	
	Model 3 (MIHS + SLJ)	0.228	0.397 [0.173, 0.621] (<0.001)	0.023 [−0.198, 0.244] (0.839)	
Cooper test (laps)	Model 1 (SLJ)	0.433	0.402 [0.176, 0.628] (<0.001)		<0.001
	Model 2 (MIHS)	0.341		0.140 [−0.051, 0.331] (0.150)	
	Model 3 (MIHS + SLJ)	0.434	0.408 [0.178, 0.638] (<0.001)	−0.016 [−0.213, 0.181] (0.873)	
20 m sprint (s)	Model 1 (SLJ)	0.602	−0.560 [−0.875, −0.245] (<0.001)		0.017
	Model 2 (MIHS)	0.484		−0.364 [−0.569, −0.159] (<0.001)	
	Model 3 (MIHS + SLJ)	0.619	−0.493 [−0.771, −0.215] (<0.001)	−0.176 [−0.334, −0.018] (0.029)	
Scratch test (cm)	Model 1 (SLJ)	0.051	0.265 [0.035, 0.495] (0.024)		<0.001
	Model 2 (MIHS)	0.009		0.077 [−0.156, 0.310] (0.517)	
	Model 3 (MIHS + SLJ)	0.051	0.275 [0.027, 0.523] (0.030)	−0.028 [−0.274, 0.218] (0.824)	

SLJ, standing long jump; MIHS, maximal isometric handgrip strength; BMI, body mass index; β, standardized beta coefficient; CI 95: confidence intervals; ΔR^2^, additional explained variance of the combined model (Model 3) relative to the best-performing single-test model (Model 1 or Model 2). In practical terms, ΔR^2^ indicates whether including both tests improves the prediction of each outcome beyond the best single test. Values close to zero suggest that one test alone is sufficient, whereas positive values indicate complementary contributions. In the present study, meaningful increases in explained variance were observed only for body mass index and, to a lesser extent, sprint performance.

## Data Availability

The data presented in this study are available on request from the corresponding author. The data are not publicly available due to ethical restrictions and privacy concerns involving minors.

## Abbreviations

The following abbreviations are used in this manuscript:

SLJ	Standing Long Jump
BMI	Body Mass Index
MIHS	Maximal Isometric Handgrip Strength
PAQ-A	Physical Activity Questionnaire for Adolescents
PSQI	Pittsburgh Sleep Quality Index
DASS–21	Depression, Anxiety, and Stress Scale–21

## References

[1] Smith J.J., Eather N., Morgan P.J., Plotnikoff R.C., Faigenbaum A.D., Lubans D.R. (2014). The health benefits of muscular fitness for children and adolescents: A systematic review and meta-analysis. Sports Med..

[2] García-Hermoso A., Ramírez-Campillo R., Izquierdo M. (2019). Is muscular fitness associated with future health benefits in children and adolescents? A systematic review and meta-analysis of longitudinal studies. Sports Med..

[3] Ortega F.B., Silventoinen K., Tynelius P., Rasmussen F. (2012). Muscular strength in male adolescents and premature death: Cohort study of one million participants. BMJ.

[4] Kettunen O., Kyröläinen H., Santtila M., Vasankari T. (2014). Physical fitness and volume of leisure time physical activity relate with low stress and high mental resources in young men. J. Sports Med. Phys. Fit..

[5] Marques A., Gomez-Baya D., Peralta M., Frasquilho D., Santos T., Martins J., Ferrari G., Gaspar de Matos M. (2020). The effect of muscular strength on depression symptoms in adults: A systematic review and meta-analysis. Int. J. Environ. Res. Public Health.

[6] Cabanas-Sánchez V., Esteban-Cornejo I., Parra-Soto S., Petermann-Rocha F., Gray S.R., Rodríguez-Artalejo F., Ho F.K., Pell J.P., Martínez-Gómez D., Celis-Morales C. (2022). Muscle strength and incidence of depression and anxiety: Findings from the UK Biobank prospective cohort study. J. Cachexia Sarcopenia Muscle.

[7] Padilla-Moledo C., Ruiz J.R., Ortega F.B., Mora J., Castro-Piñero J. (2012). Associations of muscular fitness with psychological positive health, health complaints, and health risk behaviors in Spanish children and adolescents. J. Strength Cond. Res..

[8] Zhang X., Jiang C., Zhang X., Chi X. (2022). Muscle-strengthening exercise and positive mental health in children and adolescents: An urban survey study. Front. Psychol..

[9] Bellón D., Rodriguez-Ayllon M., Solis-Urra P., Fernandez-Gamez B., Olvera-Rojas M., Coca-Pulido A., Toval A., Martín-Fuentes I., Bakker E.A., Sclafani A. (2024). Associations between muscular strength and mental health in cognitively normal older adults: A cross-sectional study from the AGUEDA trial. Int. J. Clin. Health Psychol..

[10] Evaristo S., Moreira C., Lopes L., Oliveira A., Abreu S., Agostinis-Sobrinho C., Oliveira-Santos J., Póvoas S., Santos R., Mota J. (2019). Muscular fitness and cardiorespiratory fitness are associated with health-related quality of life: Results from the LabMed Physical Activity Study. J. Exerc. Sci. Fit..

[11] Kell R.T., Bell G., Quinney A. (2001). Musculoskeletal fitness, health outcomes and quality of life. Sports Med..

[12] Marin-Jimenez N., Perez-Bey A., Cruz-Leon C., Conde-Caveda J., Segura-Jimenez V., Castro-Piñero J., Cuenca-Garcia M. (2024). Criterion-related validity and reliability of the standing long jump test in adults: The Adult-Fit project. Eur. J. Sport Sci..

[13] Ortega F.B., Cadenas-Sánchez C., Sánchez-Delgado G., Mora-González J., Martínez-Téllez B., Artero E.G., Castro-Piñero J., Labayen I., Chillón P., Löf M. (2015). Systematic review and proposal of a field-based physical fitness-test battery in preschool children: The PREFIT battery. Sports Med..

[14] Bianco A., Jemni M., Thomas E., Patti A., Paoli A., Ramos Roque J., Palma A., Mammina C., Tabacchi G. (2015). A systematic review to determine reliability and usefulness of the field-based test batteries for the assessment of physical fitness in adolescents—The ASSO Project. Int. J. Occup. Med. Environ. Health.

[15] Artero E.G., España-Romero V., Castro-Piñero J., Ruiz J.R., Jiménez-Pavón D., Aparicio V., Gatto-Cardia M., Baena P., Vicente-Rodríguez G., Castillo M.J. (2012). Criterion-related validity of field-based muscular fitness tests in youth. J. Sports Med. Phys. Fit..

[16] Bohannon R.W. (2015). Muscle strength: Clinical and prognostic value of hand-grip dynamometry. Curr. Opin. Clin. Nutr. Metab. Care.

[17] Szaflik P., Zadoń H., Michnik R., Nowakowska-Lipiec K. (2025). Handgrip strength as an indicator of overall strength and functional performance—Systematic review. Appl. Sci..

[18] Gąsior J.S., Pawłowski M., Jeleń P.J., Rameckers E.A., Williams C.A., Makuch R., Werner B. (2020). Test–retest reliability of handgrip strength measurement in children and preadolescents. Int. J. Environ. Res. Public Health.

[19] Cronin J., Lawton T., Harris N., Kilding A., McMaster D.T. (2017). A brief review of handgrip strength and sport performance. J. Strength Cond. Res..

[20] Xu J., Wan C.S., Ktoris K., Reijnierse E.M., Maier A.B. (2022). Sarcopenia is associated with mortality in adults: A systematic review and meta-analysis. Gerontology.

[21] van Sluijs E.M.F., Ekelund U., Crochemore-Silva I., Guthold R., Ha A., Lubans D., Oyeyemi A.L., Ding D., Katzmarzyk P.T. (2021). Physical activity behaviours in adolescence: Current evidence and opportunities for intervention. Lancet.

[22] Mijarra-Murillo J.J., Polo-Recuero B., Solera-Alfonso A., Arribas-Romano A., García-González M., Laguarta-Val S., Delfa-de-la-Morena J.M. (2024). Leisure time habits and levels of physical activity in children and adolescents. Children.

[23] Merikangas K.R., He J.P., Burstein M., Swanson S.A., Avenevoli S., Cui L., Benjet C., Georgiades K., Swendsen J. (2010). Lifetime prevalence of mental disorders in U.S. adolescents: Results from the NCS-A. J. Am. Acad. Child Adolesc. Psychiatry.

[24] Dong T., Wang Y., Lin Y. (2025). Prevalence and determinants of depression, anxiety, and stress among secondary school students. PLoS ONE.

[25] Yang H., Luan L., Xu J., Xu X., Tang X., Zhang X. (2024). Prevalence and correlates of sleep disturbance among adolescents in the eastern seaboard of China. BMC Public Health.

[26] Riegel B., Dunbar S.B., Fitzsimons D., Freedland K.E., Lee C.S., Middleton S., Stromberg A., Vellone E., Webber D.E., Jaarsma T. (2021). Self-care research: Where are we now? Where are we going?. Int. J. Nurs. Stud..

[27] Guzmán-Muñoz E., Mendez-Rebolledo G., Concha-Cisternas Y., Alarcón-Rivera M., Faúndez-Casanova C. (2025). Diseños de investigación cuantitativa en ciencias de la actividad física y la salud. Rev. Cienc. Act. Fís. UCM.

[28] von Elm E., Altman D.G., Egger M., Pocock S.J., Gøtzsche P.C., Vandenbroucke J.P., STROBE Initiative (2008). The strengthening the reporting of observational studies in epidemiology (STROBE) statement. J. Clin. Epidemiol..

[29] Santos C.A.F., Amirato G.R., Jacinto A.F., Pedrosa A.V., Caldo-Silva A., Sampaio A.R., Pimenta N., Santos J.M.B., Pochini A., Bachi A.L.L. (2022). Vertical jump tests: A safe instrument to improve the accuracy of the functional capacity assessment in robust older women. Healthcare.

[30] Cooper K.H. (1968). A means of assessing maximal oxygen intake: Correlation between field and treadmill testing. JAMA.

[31] Altmann S., Ringhof S., Neumann R., Woll A., Rumpf M.C. (2019). Validity and reliability of speed tests used in soccer: A systematic review. PLoS ONE.

[32] Baltaci G., Un N., Tunay V., Besler A., Gerçeker S. (2003). Comparison of three different sit and reach tests for measurement of hamstring flexibility in female university students. Br. J. Sports Med..

[33] Lavín-Pérez A.M., León-Llamas J.L., Salas Costilla F.J., Collado-Mateo D., López de Las Heras R., Gasque Celma P., Villafaina S. (2023). Validity of online supervised fitness tests in people with low back pain. Healthcare.

[34] Buysse D.J., Reynolds C.F., Monk T.H., Berman S.R., Kupfer D.J. (1989). The Pittsburgh Sleep Quality Index: A new instrument for psychiatric practice and research. Psychiatry Res..

[35] Faúndez Casanova C., Vásquez J., Souza R., Castillo M., Castillo F., Pérez J., Guzmán J. (2020). Fiabilidad y reproductividad de los cuestionarios de actividad física PAQ-C y PAQ-A en estudiantes de enseñanza básica y media de la ciudad de Talca. UCMaule.

[36] Román F., Santibáñez P., Vinet E. (2018). Uso de las escalas de depresión, ansiedad y estrés (DASS-21) como instrumento de tamizaje en jóvenes con problemas clínicos. Acta Investig. Psicol..

[37] Laakso P.T.T., Ortega F.B., Huotari P., Tolvanen A.J., Kujala U.M., Jaakkola T.T. (2024). The association of adolescent fitness with cardiometabolic diseases in late adulthood: A 45-year longitudinal study. Scand. J. Med. Sci. Sports.

[38] Vaishya R., Misra A., Vaish A., Ursino N., D’Ambrosi R. (2024). Hand grip strength as a proposed new vital sign of health: A narrative review of evidences. J. Health Popul. Nutr..

[39] Nakai Y., Usumoto Y., Takeshita Y. (2024). The effects of regional muscle strength and mass on standing long jump performance. Muscles.

[40] Wang Y., Dong D. (2023). Effects of muscle strength in different parts of adolescent standing long jump on distance based on surface electromyography. Front. Physiol..

[41] Arain M., Haque M., Johal L., Mathur P., Nel W., Rais A., Sandhu R., Sharma S. (2013). Maturation of the adolescent brain. Neuropsychiatr. Dis. Treat..

[42] Yapici H., Gulu M., Yagin F.H., Eken O., Gabrys T., Knappova V. (2022). Exploring the relationship between biological maturation level, muscle strength, and muscle power in adolescents. Biology.

[43] Bermejo-Cantarero A., Álvarez-Bueno C., Martínez-Vizcaino V., Redondo-Tébar A., Pozuelo-Carrascosa D.P., Sánchez-López M. (2021). Relationship between both cardiorespiratory and muscular fitness and health-related quality of life in children and adolescents: A systematic review and meta-analysis. Health Qual. Life Outcomes.

